# Using multiple agreement methods for continuous repeated measures data: a tutorial for practitioners

**DOI:** 10.1186/s12874-020-01022-x

**Published:** 2020-06-12

**Authors:** Richard A. Parker, Charles Scott, Vanda Inácio, Nathaniel T. Stevens

**Affiliations:** 1 Edinburgh Clinical Trials Unit, Usher Institute, University of Edinburgh, Edinburgh, UK; 2 Diabetes Trials Unit, Oxford Centre for Diabetes, Endocrinology and Metabolism, University of Oxford, Oxford, UK; 3 School of Mathematics, University of Edinburgh, Edinburgh, UK; 4 Department of Statistics and Actuarial Science, University of Waterloo, 200 University Avenue West, Waterloo, ON N2L 3G1 Canada

**Keywords:** Method comparison studies, Limits of agreement, Agreement, Concordance correlation coefficient, Repeated measures

## Abstract

**Background:**

Studies of agreement examine the distance between readings made by different devices or observers measuring the same quantity. If the values generated by each device are close together most of the time then we conclude that the devices agree. Several different agreement methods have been described in the literature, in the linear mixed modelling framework, for use when there are time-matched repeated measurements within subjects.

**Methods:**

We provide a tutorial to help guide practitioners when choosing among different methods of assessing agreement based on a linear mixed model assumption. We illustrate the use of five methods in a head-to-head comparison using real data from a study involving Chronic Obstructive Pulmonary Disease (COPD) patients and matched repeated respiratory rate observations. The methods used were the concordance correlation coefficient, limits of agreement, total deviation index, coverage probability, and coefficient of individual agreement.

**Results:**

The five methods generated similar conclusions about the agreement between devices in the COPD example; however, some methods emphasized different aspects of the between-device comparison, and the interpretation was clearer for some methods compared to others.

**Conclusions:**

Five different methods used to assess agreement have been compared in the same setting to facilitate understanding and encourage the use of multiple agreement methods in practice. Although there are similarities between the methods, each method has its own strengths and weaknesses which are important for researchers to be aware of. We suggest that researchers consider using the coverage probability method alongside a graphical display of the raw data in method comparison studies. In the case of disagreement between devices, it is important to look beyond the overall summary agreement indices and consider the underlying causes. Summarising the data graphically and examining model parameters can both help with this.

## Background

Studies of agreement examine the distance between readings made by different devices or observers measuring the same quantity. If the values generated by each device are close together most of the time such that it makes no practical difference which device is used, then we conclude that the devices agree. An example of an agreement study is when we are interested in determining the extent to which two observers using the same instrument generate similar readings. A second example is determining whether the mode of delivery of a questionnaire matters when given to the same set of participants on the same day. For example, Chen and colleagues investigated whether two different versions of the Epworth Sleepiness Scale (electronic and paper) generated the same scores when both were given to patients with obstructive sleep apnoea on the same day [1]. Since the differences between electronic and paper versions were within ± 4 most of the time, this was deemed to constitute acceptable agreement in this case [1]. Agreement has both accuracy and precision components: disagreement between devices could be due to a systematic bias of one device relative to the other, or if at least one of the devices is imprecise [2].

Several different methods for assessing the agreement of continuous data have been proposed in the literature, of which the *concordance correlation coefficient* [3, 4], and *limits of agreement* [5] methods are the most widely used. The *coverage probability* [6], *total deviation index* [6, 7], and *coefficient of individual agreement* methods [8, 9] have also been described. All five methods can be computed via linear mixed effects models. With an emphasis on practical application and interpretation, the aim of this study is to show how these five approaches can be applied to the same agreement problem and showcase the strengths and weaknesses of each method so that researchers can decide which methods to use in their own studies. Reviews of agreement indices have already been presented in the literature by Barnhart et al. (2007) [2], Obuchowski et al. (2015) [10], Barnhart et al. (2016) [11], and Barnhart (2018) [12]; with the latter three papers including real life examples to compare between agreement indices. However, the examples provided were almost exclusively sourced from the fields of quantitative imaging and core laboratory research. In this article we extend the methodological work already accomplished to the area of analysing clustered unbalanced data in applied clinical research, specifically in the area of measuring respiratory rate in patients with COPD. Furthermore, we focus specifically on the linear mixed effects model implementation of the methods rather than the more general approach used in the aforementioned papers. For limits of agreement in particular, this implementation of the method is not considered in previous reviews. The justification of this focus is because mixed effects modelling is increasingly used in clinical research and has advantages over fixed effects methods (e.g. Analysis Of Variance (ANOVA)) for several reasons outlined in Brown (2015) [13]. In particular, (i) missing or unbalanced data poses fewer problems for analysis, and (ii) inference can be made based on a wider population of patients [13]. We also focus on agreement problems with repeated observations because these are recommended when assessing agreement [14]. Finally, to help practitioners of agreement methods, we have also provided the R code needed to implement the methods in a Supplementary Materials file.

The agreement problem investigated in this paper originates from a study in COPD patients which we describe in more detail below. As such, our focus is on clustered and unbalanced designs: that is, repeated measures data for which the number of observations per cluster may not be the same, and for which there may be multiple levels of clustering. Here we treat subjects as clusters. Such data structures are common in medical research due to necessary observational designs and missing data. Most of the methods rely on parametric assumptions, although other approaches are possible which do not require these assumptions and are mentioned briefly below.

## Methods

### Illustrative example

By means of an illustrative example, we compare and contrast the five different agreement methods mentioned before and provide guidance for selecting among them. Our example consists of respiratory rate measurements (in breaths per minute) from 21 subjects with COPD, which were measured simultaneously by six devices (including a gold standard device) worn at the same time. This was the dataset used in the study by Parker and colleagues [15], and has been made publicly available via data sharing [15]. Multiple time-matched respiratory rate measurements were taken on each patient, so there was clustering of repeated observations by participant. Eleven different activities were performed by participants during a laboratory-based protocol that was 57 min in duration. These were sitting, lying, standing, slow walking, fast walking, sweeping, lifting objects, standing and walking, climbing stairs, treadmill (flat walking), and treadmill (4% slope). The balance of activities was chosen to be representative of the activities encountered in daily life [16]. Not everyone performed exactly the same number of activities because some tasks were too difficult for some participants (e.g. the treadmill), and so this is an example of an unbalanced study design. Most activities had just one respiratory rate reading per participant, but “sitting” and “standing and walking” had 6–7 and 1–3 observations per participant, respectively (see Figure 1 in the Supplementary File), and therefore there was clustering of observations within activities as well as within participants. Eight of the participants (38%) were female, with an overall mean age of 69 (Standard Deviation (SD) 8) and mean Body Mass Index (BMI) of 26 (SD 6). Full details about the study are given elsewhere [16]. For simplicity, in this article, we only consider the comparison of one of the devices (chest-band) with the gold standard device (Oxycon mobile, Carefusion). Among the six devices used in the study, the chest-band device and the gold standard were the only two devices which had no missing data. The chest-band device was also one of the devices which showed the best agreement with the gold standard.

### Terminology

In what follows, the five statistical methods for assessing agreement with repeated measures data are described in turn with corresponding model formulae. As described above, linear mixed effect models are particularly appropriate for analysing data from clustered and unbalanced designs because they incorporate random effect terms. The basic linear mixed model is of the form:

1 $$ {y}_{ijlt}=\mu +{\alpha}_i+{\beta}_j+{\gamma}_l+{\varepsilon}_{ijlt} $$

where *y*_*ijlt*_ represents the respiratory rate reading/measurement made on subject *i* by device *j* when performing activity *l* at time *t*; *μ* is the overall mean; ${\alpha}_i\sim N\left(0,{\sigma}_{\alpha}^2\right)$ is the random subject effect; *β*_*j*_ is the fixed effect of the device which, for identifiability reasons, we require *β*_1_ + *β*_2_ = 0; ${\gamma}_l\sim N\left(0,{\sigma}_{\gamma}^2\right)$ denotes the random activity effect, and ${\varepsilon}_{ijlt}\sim N\left(0,{\sigma}_{\varepsilon}^2\right)$ is the residual error. We extend and modify this basic model for each of the specific agreement methods below. In other settings, “device” may refer to “systems”, “raters”, “methods”, “instruments” or “observers”. Likewise, “subject” may refer to “participant”, “patient”, “site”, “experiment”, “mode” in other settings. In the COPD example, the *y*_*ijlt*_ are time-matched repeated measurements collected by each device on each subject. For the limits of agreement method, the linear mixed model is instead fitted to “paired differences” denoting the between-device differences measured at exactly the same time in each subject.

### Model assumptions

In what follows, the five statistical methods for assessing agreement with repeated measures data are described in turn. The five main methods are all based on linear mixed effects models, and so they rely on similar (if not identical) model assumptions. Logically if the mixed model assumptions are not valid, then neither is the agreement index calculated on the basis of this model. See Table 1 for a list of common model assumptions and techniques that may be used to evaluate them.

**Table 1:** Standard agreement model assumptions (with suggested procedures to check their validity in brackets)

• Independent subjects	
• Normally distributed random effects (diagnosed by Q-Q plots)	
• Normally distributed error terms (diagnosed by Q-Q plots)	
• Fixed mean bias across the range of measurement (plots of standardized residuals against fitted values)	
• Constant between-subject and within-subject variabilities across the range of measurements (plots of residuals against fitted values)	

### Concordance correlation coefficient for repeated measures

The concordance correlation coefficient (CCC) method was developed by Lin in 1989 [3], with the longitudinal repeated measures version of the CCC developed by King et al. [4], Carrasco et al. [17] and Carrasco and Jover [18]. The CCC is a standardized coefficient taking values from − 1 to 1, where 1 indicates perfect agreement and − 1 indicates perfect disagreement. For the CCC model, the individual readings are modelled using a combination of random effects and fixed effects. Interaction terms are often also included. In particular, in the context of our COPD example, we assume the following linear mixed effects model

2 $$ {y}_{ij lt}=\mu +{\alpha}_i+{\beta}_j+{\gamma}_l+{\left(\alpha \beta \right)}_{ij}+{\left(\alpha \gamma \right)}_{il}+{\left(\beta \gamma \right)}_{jl}+{\varepsilon}_{ij lt} $$

where *y*_*ijlt*_ represents the respiratory rate reading/measurement made on subject *i* by device *j* when performing activity *l* at time *t*; *μ* is the overall mean; ${\alpha}_i\sim N\left(0,{\sigma}_{\alpha}^2\right)$ is the random subject effect; *β*_*j*_ is the fixed effect of the device (as before, we assume that *β*_1_ + *β*_2_ = 0); and ${\gamma}_l\sim N\left(0,{\sigma}_{\gamma}^2\right)$ denotes the random activity effect. Further, (*αβ*)_*ij*_, (*αγ*)_*il*_, and (*βγ*)_*jl*_ denote, respectively, the random interaction between subject and device, between subject and activity, and between device and activity and we follow the usual assumption that they are normally distributed with mean zero and with variance ${\sigma}_{\alpha \beta}^2,{\sigma}_{\alpha \gamma}^2,$ and ${\sigma}_{\beta \gamma}^2$, respectively. Finally, ${\varepsilon}_{ijlt}\sim N\left(0,{\sigma}_{\varepsilon}^2\right)$ is the error. All random effects are assumed to be independent.

We justify these modelling choices as follows. In line with Parker et al. [15] we regard subjects as random effects, therefore implicitly assuming they are a sample from a wider population of COPD patients (rather than treating them as consisting of the entire population of interest); this maximises generalisability of the results to the true population of interest (i.e. all COPD patients). We regard activity as a random effect as well, mainly so that we can generalize the results to any activity from a wider “population” of activities performed by participants in daily life, but also so that activities with small numbers of respiratory rate readings are not weighted too highly in the model (i.e. shrinkage causes the effect of individual activities to be drawn towards the population-averaged effect). All possible two-way interactions were included in the model and they take into account the variability in subjects across devices, in subjects across activities, and in devices across activities. In this example, it was not expected that the respiratory rate measured under the same device, activity and subject would change at different measurement times, and so the time ordering of measurements was not deemed to be relevant. We therefore treat all measurements taken under the same device, activity and subject as replications, and assume *y*_*ijlt*_ to be identically and independently distributed given subject, device and activity.

Under the assumption of a fixed device effect, Carrasco et al. [17] showed that the CCC for repeated measurements coincides with the Intra-class Correlation Coefficient (ICC) and as such it can be written as

$$ {\rho}_{CCC}=\frac{Cov\left({y}_{i1 lt},{y}_{i2 lt}\right)}{Var\left({y}_{ijlt}\right)}=\frac{\sigma_{\alpha}^2+{\sigma}_{\gamma}^2+{\sigma}_{\alpha \gamma}^2}{\sigma_{\alpha}^2+{\phi}_{\beta}^2+{\sigma}_{\gamma}^2+{\sigma}_{\alpha \gamma}^2+{\sigma}_{\alpha \beta}^2+{\sigma}_{\beta \gamma}^2+{\sigma}_{\varepsilon}^2} $$

Note that the variance due to the random part is ${\sigma}_{\alpha}^2+{\sigma}_{\gamma}^2+{\sigma}_{\alpha \gamma}^2+{\sigma}_{\alpha \beta}^2+{\sigma}_{\beta \gamma}^2+{\sigma}_{\varepsilon}^2$ and the variance due to the fixed factor (device) is ${\phi}_{\beta}^2={\sum}_{j=1}^2{\beta}_j^2$, which accounts for the systematic differences between the two devices. If this latter term is not included, one is measuring consistency between devices rather than their agreement. The total variance is then ${\sigma}_{\alpha}^2+{\phi}_{\beta}^2+{\sigma}_{\gamma}^2+{\sigma}_{\alpha \gamma}^2+{\sigma}_{\alpha \beta}^2+{\sigma}_{\beta \gamma}^2+{\sigma}_{\varepsilon}^2.$

The CCC, in this particular case, thus reflects the proportion of the total overall variability explained by the subject and activity effects (and their interaction) and a CCC of 1 implies that there is no variability in the device across subjects and activities.

### Mixed effects limits of agreement

Bland and Altman first proposed the limits of agreement (LoA) method over 30 years ago in their 1986 paper [5] as an alternative to correlation-based methods which they believed did not accurately characterize agreement [19]. The 95% limits of agreement are simply calculated as m ± 2 *SD, where *m* is the mean of the paired differences in readings (e.g. differences in respiratory rate measured at the same time in the same participant using two different devices) and *SD* is the standard deviation of the paired differences. The limits of agreement are meant to quantify dispersion among the paired differences. The wider the limits of agreement, the more dissimilar the devices’ readings are expected to be, suggesting a lack of agreement between devices. To formally judge this level of agreement, the limits are compared to a clinically acceptable difference (CAD): a range within which differences are considered practically negligible. If the limits are contained within the range of the CAD then it is concluded that the devices agree and could be used interchangeably. The CAD should be decided before data analysis to avoid any bias in the decision, though strictly speaking the statistical validity of the method does not require this. The limits of agreement are typically shown overlaid on a Bland-Altman plot of the paired differences against the averages of the paired readings.

In the repeated measures case, applying the standard limits of agreement to the data will result in limits that are too narrow because they do not take into account the reduction in variability that arises when working with averages of readings. In this case we need to use a specially adapted version of the limits of agreement, for which there are several methods available. Bland and Altman first described a fixed effects ANOVA method to extend the LoA method to account for repeated measures [20] and this method is succinctly described in the Supplementary Materials.

There have also been a diverse range of mixed effects models proposed that vary in complexity as a means to quantify dispersion in differences and hence calculate limits of agreement. Some of these models are similar to the CCC in that they model the raw outcome data and include interaction terms; while other authors suggest modelling the differences directly [15, 21–24]. The relatively simple methodology that Parker et al. [15] recommend, and that we adopt here (see Eq. (3)), directly models the differences through a linear mixed effects model, and is highly adaptable to different data structures. Indeed, the methodology has the flexibility and versatility to accommodate complex variability structures [25, 26].

For our COPD motivating example, and letting *D*_*ilt*_ be the difference between the readings made by the two devices when subject *i* is performing activity *l* at time *t*, i.e., *D*_*ilt*_ = *Y*_*i*2*lt*_ − *Y*_*i*1*lt*_, we model these paired differences through the following linear mixed effects model

3 $$ {\displaystyle \begin{array}{c}{D}_{ilt}={\mu}^{\ast }+{\alpha_i}^{\ast }+{\gamma_l}^{\ast }+{\varepsilon}_{ilt}^{\ast}\\ {}{\alpha_i}^{\ast}\sim N\left(0,{\sigma}_{\alpha^{\ast}}^2\right),{\gamma_i}^{\ast}\sim N\left(0,{\sigma}_{\gamma^{\ast}}^2\right),{\varepsilon}_{ilt}^{\ast}\sim N\left(0,{\sigma}_{\varepsilon^{\ast}}^2\right)\end{array}} $$

where *μ*^∗^ is the overall mean of the between-device differences, *α*_*i*_^∗^ is the random effect of the *i*^th^ subject, *γ*_*l*_^∗^ is the random effect of the *l*^th^ activity, and ${\varepsilon}_{0 ilt}^{\ast }$ is the error term. We use asterisks to distinguish these quantities from their counterparts in model (1) which is defined in terms device readings directly (as opposed to their differences). In order to generate an appropriately weighted estimate of the mean bias, Parker et al. [15] proposed to fit a separate regression model only including a constant term and a random effect for subjects (i.e., without considering activity), that is

$$ {D}_{ilt}={\mu}_0^{\ast }+{\alpha}_{0i}^{\ast }+{\varepsilon}_{0 ilt}^{\ast } $$

$$ {\alpha}_{0i}^{\ast}\sim N\left(0,{\sigma}_{\alpha_0^{\ast}}^2\right),{\varepsilon}_{0 ilt}^{\ast}\sim N\left(0,{\sigma}_{\varepsilon_0^{\ast}}^2\right) $$

where ${\mu}_0^{\ast }$ is the mean bias of interest. The limits of agreement are then calculated as

$$ {\mu}_0^{\ast}\pm 1.96\sqrt{\sigma_{\alpha^{\ast}}^2+{\sigma}_{\gamma^{\ast}}^2+{\sigma}_{\varepsilon^{\ast}}^2} $$

with the square root of the total variance giving an estimate of the standard deviation for use in the conventional Bland-Altman limits of agreement formula.

It is worth remarking that the limits of agreement can also be calculated from the model in Eq. (2), which leads to the following expression for the paired differences

$$ {D}_{ilt}^{\ast }={y}_{i2 lt}-{y}_{i1 lt}=\left({\beta}_2-{\beta}_1\right)+\left[{\left(\alpha \beta \right)}_{i2}-{\left(\alpha \beta \right)}_{i1}\right]+\left[{\left(\beta \gamma \right)}_{2l}-{\left(\beta \gamma \right)}_{1l}\right]+\left({\varepsilon}_{i2 lt}-{\varepsilon}_{i1 lt}\right) $$

The mean bias is then quantified by (*β*_2_ − *β*_1_) and further $Var\left({D}_{ilt}^{\ast}\right)=2{\sigma}_{\alpha \beta}^2+2{\sigma}_{\beta \gamma}^2+2{\sigma}_{\varepsilon}^2$ and, therefore, the limits of agreement are computed as

$$ {\beta}_2-{\beta}_1\pm 1.96\sqrt{2{\sigma}_{\alpha \beta}^2+2{\sigma}_{\beta \gamma}^2+2{\sigma}_{\varepsilon}^2} $$

The benefit of using model (3) is that the normality assumption is more likely to be valid if it is based on the differences. In particular, it is possible that the differences follow a normal distribution even if the raw measurements do not, but the converse is not true.

### Coverage probability

The limits of agreement approach seeks to determine whether the differences between devices are small enough, on average, to be considered clinically acceptable. This is determined by evaluating whether their limits of variation are contained within the interval of clinically acceptable differences. The coverage probability (CP) proposed by Lin et al. [6] answers this same question more directly by calculating the probability that the between-device differences themselves lie within the boundary of some tolerance interval – what Bland and Altman refer to as the range of clinically acceptable differences. Clearly, larger probabilities indicate closer agreement. In practice the researcher must decide whether the CP value is large enough to use the two devices interchangeably.

To calculate the CP in practice for our COPD example we first use the linear mixed effects model in (2) to calculate the mean squared deviation which is the expected squared difference between readings by two different devices on the same individual performing the same activity at the same time:

$$ MSD\left({Y}_1,{Y}_2\right)=E\left\{{\left({Y}_{i1 lt}-{Y}_{i2 lt}\right)}^2\right\}={\left({\beta}_1-{\beta}_2\right)}^2+2\left({\sigma}_{\alpha \beta}^2+{\sigma}_{\beta \gamma}^2+{\sigma}_{\varepsilon}^2\right) $$

Second, the CP is computed as

$$ CP\left(\delta \right)=1-2\left\{1-\varPhi \right(\delta /\sqrt{MSD\left({Y}_1,{Y}_2\right)}\Big\} $$

where ±δ is the range of clinically acceptable differences and Φ(·) is the standard normal cumulative distribution function.

### Total deviation index

The total deviation index (TDI) [6, 7] is closely related to the coverage probability. For the CP, one must pre-specify the range of clinically acceptable differences and then the probability of containment is calculated. The TDI, on the other hand, reverses this process; for a given containment probability *p* the TDI calculation provides the boundary within which the differences will be contained *p* × 100% of the time. This approach is useful in situations when specifying a CAD is difficult or impossible. The practitioner must then decide whether the calculated boundary is narrow enough for the devices to be used interchangeably. For our COPD example, under the assumptions of model (2), the TDI can be written as

$$ TDI(p)={\varPhi}^{-1}\left(\left(1+p\right)/2\right)\sqrt{MSD\left({Y}_1,{Y}_2\right)} $$

where *p* is the pre-specified proportion of between-device differences that we hope to be contained within the interval ±δ.

### Coefficient of individual agreement

The Coefficient of Individual Agreement (CIA) was developed by Haber and Barnhart [8] and Barnhart et al. [9]. It is a scaled coefficient which directly compares the disagreement *between-devices* to the disagreement *within-devices* within subjects [27, 28]. Essentially, the CIA attempts to quantify by what magnitude the variability between different devices increases when compared to the replication variability within devices. The value of the CIA ranges from 0 to 1, with 1 indicating that using different devices makes no difference to the variability of repeated measurements taken under the same conditions within the same subject. The residual error variance ${\sigma}_e^2$ represents the variability of repeated measurements taken under the same conditions within the same subject and therefore it is important that this is a reliable benchmark for comparison. As recommended by others [27, 28], we check that this value is reasonable by calculating the repeatability coefficient of Bland and Altman, which is $1.96\sqrt{2{\sigma}_e^2}$. There are different variants of the CIA, but we follow others in using the mean squared deviation as the disagreement metric [27, 28]. In particular, we follow the approach for matched repeated measures outlined in Haber et al. [28], which suggests that calculation of the CIA should be based on

$$ CIA=\frac{MSD\left({Y}_j,{Y}_j^{\prime}\right)}{MSD\left({Y}_1,{Y}_2\right)} $$

The term $MSD\left({Y}_j,{Y}_j^{\prime}\right)$ denotes the mean squared deviation between two (hypothetical) replicated readings, *Y*_*j*_ and ${Y}_j^{\prime }$, that could be made by device *j* on the same subject under the same activity at the same time. In our COPD context, and assuming model (2) for the respiratory rate measurements, we have

$$ CIA=\frac{2{\sigma}_{\varepsilon}^2}{{\left({\beta}_1-{\beta}_2\right)}^2+2\left({\sigma}_{\alpha \beta}^2+{\sigma}_{\beta \gamma}^2+{\sigma}_{\varepsilon}^2\right)} $$

### Alternative methods

Stevens et al. [14, 29] developed the probability of agreement (PoA) method as an alternative to the limits of agreement approach, which has the advantage of taking into account two different types of bias and unequal precisions across devices. Proportional bias, where the magnitude of disagreement depends on the true value in each subject, is considered in addition to additive bias, and this information can be used to elucidate the different sources of disagreement if the devices do not agree. The PoA method provides a flexible and informative summary of agreement, but at present the methodology does not adjust for confounders (e.g. activity in our COPD study) and so it is not yet as widely applicable as other alternatives. Further details about this method are provided in the Supplementary File.

If the assumptions described above are not valid, then non-parametric methods should be considered. For example, Perez-Jaume and Carrasco suggest a non-parametric alternative to calculate the TDI which is more stable and reliable than the parametric method when working with skewed data [30]. It is also relatively simple to calculate and less influenced by outliers or extreme values than the parametric approach. The method involves simply calculating quantiles of an ordered list of paired differences to calculate the TDI. A bootstrap method can then be used to calculate the upper bound by resampling at the patient level and then recalculating the TDI for each bootstrap resample. This appears to be the same as a percentile method first described by Bland and Altman [5], except that in the repeated measures case we use bootstrap resampling to obtain the upper bound. Although it does not assume a normal distribution, we still need to assume that the paired differences are independent and identically distributed. Other non-parametric methods are available [31, 32]. Stevens [33] has also developed a generalization of the probability of agreement based on the method of moments that does not require any distributional assumption for the true values. Fully Bayesian versions of the limits of agreement method have also been proposed, for example Schluter’s Bayesian agreement method [34]. Additionally, Barnhart [12] and Barnhart et al. [11] describe an interesting method involving the use of generalised estimating equations to provide a non-parametric estimate of the CP. Recently Jang et al. [35] have proposed a new set of agreement indices suitable for contexts in which there are multiple raters and heterogeneous variances.

Besides the methods mentioned above, other methods have been used to assess agreement, although some of these are inappropriate. A systematic review [36] of agreement studies that were reported between 2007 and 2009 found that around 10% of studies were using inappropriate methods to assess agreement including standard correlation coefficients, the coefficient of determination from a regression analysis (R-squared), and comparison of means methods (e.g. t-tests to detect mean differences).

In the repeated measures case, aggregation methods have been used whereby summary statistics are computed at the subject level in order to reduce the dependence in the data. Although aggregating data to the patient level works in some studies with repeated measures, it is usually not appropriate in the agreement context because the variability within subjects is often of primary interest and we would be losing important information by aggregating.

Another method seen in the literature involved first performing a statistical test to determine if the clustering was important and then if not, carrying out an analysis without adjusting for clustering [37]. This method is not recommended because even if the test for clustering is statistically non-significant, the clustering in the data may still be sufficient to bias the agreement index.

## Results

**Fig. 1:**
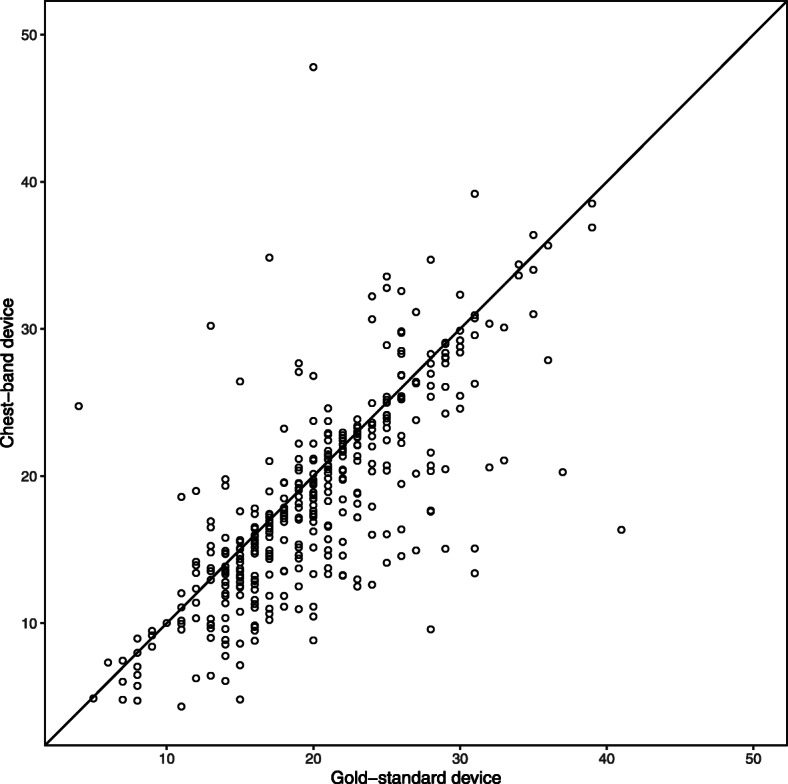
Scatterplot comparing the chest-band and gold standard measurement devices. Each point corresponds to individual measurements made on a subject. The solid diagonal line is the line of equality

**Fig. 2:**
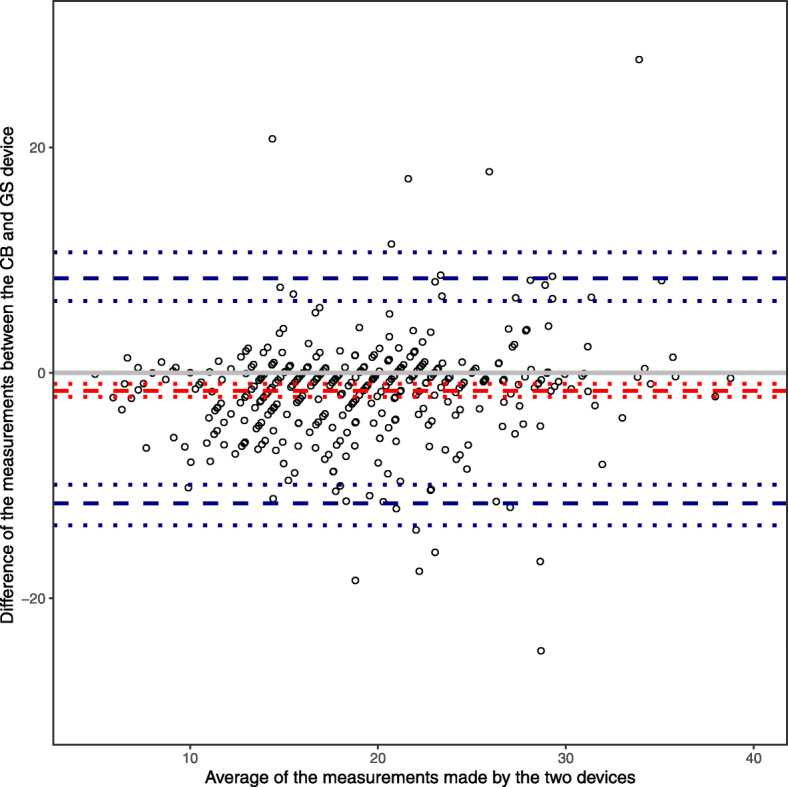
Bland-Altman plot showing the paired difference between the devices against the average of the pairs of devices. Points shown correspond to individual pairs of observations rather than individual patients. Dashed line shows the mean bias (red) and limits of agreement (blue). Dotted lines are 95% bootstrap confidence intervals

Twenty-one patients with COPD each provided a mean of 18 paired readings on the chest-band and gold-standard devices (median 19, range 15–19), with 16 patients recording the maximum of 19 readings across the different experimental activities. As already reported elsewhere [16], the participants had a mean age of 69 (SD 8), with mean BMI of 26 (SD 6), and 13 (62%) were men. The median respiratory rate was 20 breaths per minute (interquartile range (IQR), 16 to 24) using the gold standard device and 18 breaths per minute (IQR 14 to 23) for the chest-band. To supplement these descriptive statistics, we provide a few exploratory plots that summarize the data in the Supplementary Material. In this supplement, Figure 1 shows the frequency of each of the 11 activities over the 21 participants, whereas Figure 2 displays a boxplot of the respiratory rate measurements for each activity, when measured by the gold standard and chest-band devices, respectively.

For the comparison of respiratory rates between the chest-band and gold standard devices, naïve estimates of agreement (which do not take clustering into account) were computed to provide simple and quick summaries of agreement: Pearson’s correlation coefficient was 0.74 (95% confidence interval (CI) 0.69 to 0.78), the concordance correlation coefficient was 0.72 (95% CI 0.67 to 0.76), and simple limits of agreement were from − 6.40 to 3.19 with a mean bias of − 1.61.

**Fig. 3:**
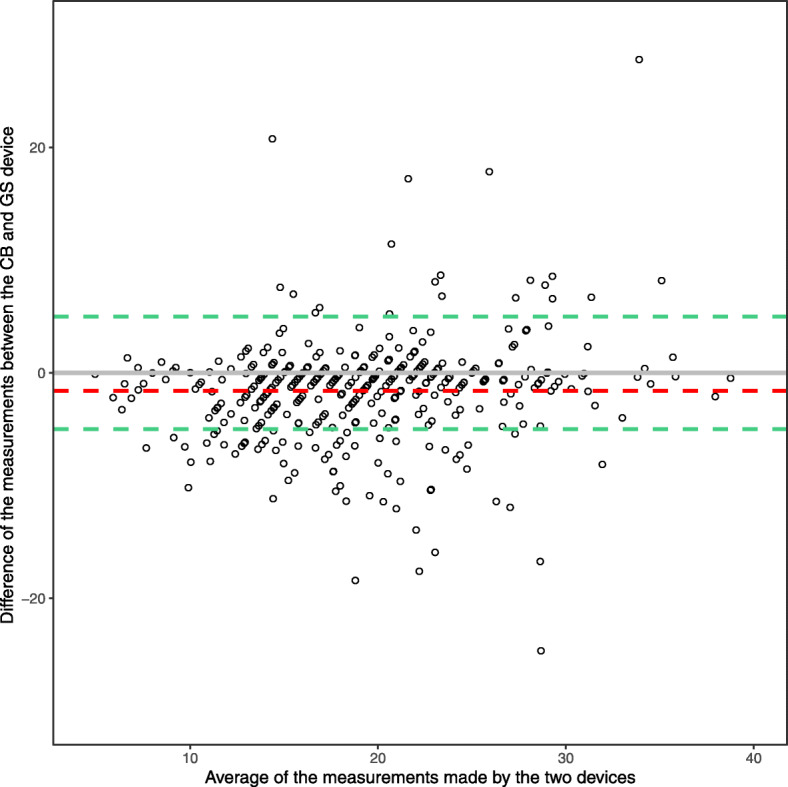
Bland-Altman style plot corresponding to the calculation of the coverage probability showing the raw data, raw mean bias (red), and clinically acceptable difference of 5 (green)

When taking into account repeated measures per subject, we began by fitting model (2) to the COPD data with the aid of the lmer function from the R package lme4 [38, 39]. Diagnostic plots are presented in Figures 3 and 4 of the Supplementary Material. The variance component estimates are as follows: ${\sigma}_{\alpha}^2=11.4,{\sigma}_{\gamma}^2=16.6,{\sigma}_{\alpha \beta}^2=0.4,{\sigma}_{\alpha \gamma}^2=6.0,{\sigma}_{\beta \gamma}^2=3.7$, and ${\sigma}_{\varepsilon}^2=10.5.$ Activity and subject do explain a considerable proportion of the overall variance, and therefore are the main sources of disagreement. The subject-device interaction is negligible, indicating no evidence of a difference in the device effect across subjects.

The concordance correlation coefficient was estimated to be 0.68 (95% CI 0.60 to 0.72). All confidence intervals were obtained through a bootstrap procedure (at the individual level). The CCC is positive and the confidence interval does not include zero or negative values, indicating that the chest band device is in *slight* agreement with the gold-standard device. A value of the CCC of 0.68 may constitute acceptable agreement, but investigators would have to agree beforehand what CCC value is required to conclude that the devices can be used interchangeably. Note that although this CCC does not differ much from the one that ignores the repeated measured nature of the data, the 95% confidence intervals, as expected, do differ by a considerable extent. Although the CCC is not a graphical method, certain graphs can complement the numerical results. For example, a scatterplot of the observations from each device plotted against each other, with a line superimposed on the plot showing the line of perfect agreement (i.e. with intercept 0 and slope 1) (see Fig. 1). Or a Bland-Altman plot could be used which involves plotting the between-method differences against the average (Fig. 2).

When applying the mixed effects limits of agreement method to the COPD data via model (3), we calculated a mean bias of − 1.60 (95% LoA − 11.57 to 8.38). The results when using model (2) are − 1.28 (95% LoA − 11.86 to 9.30). The results when using only fixed effects were: mean bias of − 1.61 (95% LoA − 9.99 to 6.78) [15]. Note that these limits of agreement are all much wider than the naïve estimates which ignored clustering. This may be because within-subject variability is treated as between-subject variability in estimating naïve LOAs, which leads to biased intervals, and illustrates the importance of taking clustering into account. Note also that the raw mean bias is very similar to the random effects mean bias in our case, and simply calculating the raw mean bias with 95% LoA calculated from mixed effects model is an acceptable alternative [15]. The CAD was set to be ± 5 based on investigators’ clinical judgement; any differences less than 5 breaths per minute were regarded as clinically unimportant. Since the limits of agreement lie outside the CAD we conclude that the two devices do not show the desired level of agreement. Figure 2 shows the corresponding Bland-Altman plot with LoA overlain. Based on the LoA model, the between-subject variance of the differences was only 0.96 compared to 7.57 for the between-activity variance of the difference. The residual variance of the LoA model (within-subject and activity variance) was 17.37.

Regarding the coverage probability, if we take *δ*= 5 to be the pre-specified boundary (CAD =± 5), the coverage probability is only 0.63 (95% CI 0.56 to 0.70), indicating relatively poor agreement between methods. This is well below the 0.95 threshold we were using to denote satisfactory agreement.

Based on a pre-specified proportion of *p* = 0.95 for containing the between-device differences, the 95% TDI was calculated to be 10.9 (95% CI 9.4 to 12.7), based on a mean-squared deviation of 30.8 (95% CI 23.0 to 41.7). This suggests that differences between the chest band and the gold-standard readings are expected to lie within ± 10.9 95% of the time. In general, whether this interval is narrow enough to signify agreement must be determined by the researcher. For these data (where the CAD is ± 5) it is clear that the TDI is too large to conclude that the two devices should be used interchangeably. Note that the TDI limits are similar to those implied by the LoA.

Before applying the Coefficient of Individual Agreement method to the COPD data, we check that the residual error variance is reasonable by calculating the repeatability coefficient of Bland and Altman, which is $1.96\sqrt{2{\sigma}_e^2}$ =8.98 when applied to the COPD data. This tells us that there is approximately 95% probability that the repeated respiratory rate values are within 9 breaths per minute of each other. In the study context, below 5 is ideal, so the repeatability coefficient is unacceptably high in this context. This means we should be cautious about over interpretation of the CIA results because they are compared against a high benchmark. The CIA was calculated to be 0.68 (95% CI 0.56 to 0.70). It has been suggested that agreement is only considered “acceptable” if the CIA exceeds 0.8 [8, 27, 28]; or in other words, if the disagreement between devices is within 25% of the level of disagreement of the repeated measurements within devices and within patients. Therefore, the CIA results suggest poor agreement between the devices, in keeping with results from the other methods. From the variance component estimates of model (2) we can elucidate the main sources of disagreement. There is substantial variability due to subjects and activities (${\sigma}_{\alpha}^2=11.4,{\sigma}_{\gamma}^2=16.6$) which may be the reason why in the CCC we have concluded that the chest-band device is in *slight* agreement with the gold standard device. Importantly however, the within-subject residual is high (${\sigma}_{\varepsilon}^2=10.5\Big)$ and the device-activity interaction is moderate $\left({\sigma}_{\beta \gamma}^2=3.7\right)$, which have contributed to our conclusion that the agreement between the two devices is not satisfactory for the CP, TDI, and CIA methods. The relatively large variability of activity and subject does not play a role in the calculation of the CP, TDI and CIA, and so this may explain the difference in conclusion compared to the CCC.

On the basis of the investigations described above, each of the five statistical approaches is summarised in Table 2. Further statistical details associated with these methods, additional diagnostic plots, and the R code used to produce the results are all provided in the Supplementary Material.

**Table 2:** Summary of the different statistical approaches

Statistical Approach	Advantages/Strengths	Disadvantages	Key summary results (COPD study example)
Concordance correlation coefficient	- A widespread and frequently used method. - Can still be used in cases where defining an appropriate CAD is either very difficult or impossible.	- Heavily influenced by the degree of between-subject and between-activity variability and the range of the data. - Can be very difficult to determine if the CCC is large enough to constitute acceptable agreement. - Can be very difficult to interpret clinically: interpretation not in terms of original measurement unit.	CCC 0.68 (95% CI 0.60 to 0.72)
Limits of agreement	- Simplicity of application: relatively straightforward to compute limits. - Clinical interpretation is based on the original measurement scale. - Estimate of mean bias. - Easy to understand and interpret.	- Standard approach is highly dependent on the normality assumption for validity. - High variability in residual errors may mask the fact that a device could measure the true value more precisely than the gold-standard. - Easy to apply method incorrectly without explicitly specifying a clinically acceptable difference.	Mean bias −1.60 95% LoA − 11.57 to 8.38
TDI	- Easy to compute. - Easy to interpret. - Clinical interpretation is based on the original measurement scale.	- Can be difficult to determine if the TDI is large enough to constitute acceptable agreement. - Does not explicitly calculate the mean bias.	TDI 10.9 (95% CI 9.4 to 12.7)
CP	- Easy to interpret. - Easy to compute. - Method cannot be used without explicitly specifying a clinically acceptable difference, which is based on the original measurement scale.	- Does not explicitly calculate the mean bias.	CP of 0.63 (95% CI 0.56 to 0.70) for boundary of ± 5
CIA	- Directly compares the disagreement between devices against the disagreement within devices and within subjects. - Much less dependent on the between-subject and between-activity variability compared to the CCC. - Can still be used in cases where defining an appropriate CAD is either very difficult or impossible.	- Depends heavily on the within-subject within-device variance. - Relies on data which has acceptable replication error.	CIA 0.68 (95% CI 0.57 to 0.75)

## Discussion

There is a plethora of methods available to assess continuous agreement in the literature which vary in complexity and in their underlying assumptions. In this article we have surveyed five different methods to analyse the same agreement problem involving clustered and unbalanced data; including some which are well known and frequently applied in the literature, and others which encompass recent advances in agreement research.

As applied to an example in COPD, we showed how all five of the agreement indices can be derived from the same linear mixed effects model (although for the LoA method we favoured a slightly different linear mixed effects model based on the paired differences). It was not surprising therefore that all five methods provided similar results, although the lack of acceptable agreement was clearer with some methods than others due to the way the variance components entered into the expression of the different agreement indices. The 95% LoA ranged from − 12 to 8 breaths per minute, and the TDI was estimated to be 11 breaths per minute, which were all well outside the clinically acceptable difference (CAD) of 5 breaths per minute. The CP was also low at 0.63 based on a CAD of 5. By examining the variance components of the LoA model (3), we observe that the between-subject variability of the paired differences was very low, but the within-subject variability and variability due to activities were both relatively high and these were the driving force behind the disagreement. Similarly, based on the variance components of model (2), we observe that the residual error variability and activity-device interaction were both reasonably high. We can infer therefore that the chest-band device may be less able to accurately capture changes in breathing rate as it varies across different activities compared to the gold standard.

One of the main ways of classifying the different methods is to divide them into those that produce standardized agreement indices that are scaled to be within a certain range (e.g. the CCC is scaled to be between − 1 and 1 and the CIA between 0 and 1), and those that allow direct comparison to the original scale of the data and require the specification of a clinically acceptable difference (e.g. the LoA, CP and TDI methods). These groups of methods are commonly referred to as scaled and unscaled agreement methods respectively [2], and the latter set of methods are sometimes known as “pure agreement indices” [40]. Indeed, the CCC can be more accurately described as assessing distinguishability rather than agreement, since it is designed to calculate the proportion of the variance of a system explained by the subject/activity effect, and does not require a CAD to be specified [41]. It is therefore not a “pure agreement index” [41]. The CCC has the disadvantage of being heavily dependent on the between-subject variability (and in our case also on the between-activity variability) and would therefore attain a high value for a population with substantial heterogeneity between subjects or activities even though the agreement within subjects might be low [2, 11, 12]. Similarly, if both the between subject and between-activity variances are very low, then the CCC is unlikely to attain a high value even if agreement within devices is reasonable. Moreover, as for the intra-class correlation coefficient (ICC), it is not related to the actual scale of measurement or to the size of error which might be clinically allowable, which makes interpretation difficult [41]. As outlined in other papers [11, 12, 40], it is very easy to obtain an artificially high value of CCC and manipulation of the dataset can change the estimate of the CCC drastically. Nevertheless, the variance components are automatically generated in R which helps one to interpret the overall summary indices.

Barnhart et al. [42] discuss how the CIA compares to the CCC in assessing agreement. They recommend using the CIA if the within-subject variability is acceptably low, particularly if the between-subject variability is large relative to the within-subject variability [42]. This is because the CIA has the distinct advantage of being less dependent on the between-subject variability than the CCC, and so is preferable to the CCC in many cases. Moreover, the CIA is expressed conditional on any confounders (e.g. the effect of time or activity) as well as being conditional on the subject effect and therefore has intuitive appeal. However, interpretation of the CIA may be challenging because it is not based on the original unit of measurement.

In contrast, the limits of agreement and TDI methods have the advantage of being based on the original unit of measurement and can be compared against a clinically acceptable difference [43]. In reviews by Barnhart et al. [11] and Barnhart [12], the authors highlight that for the LoA, it is possible to have 95% of the differences within the clinically acceptable difference but yet not conclude agreement (if, for example, one of the limits is outside the CAD). This can happen with skewed data or because of some other failure of the normality assumption. We agree that this may be an issue when seeking to interpret the LoA and that checking of assumptions when performing LoA is particularly important. However, we think the ability of the methodology (and the Bland-Altman plot in particular) to reveal relative mean biases, patterns in the data and hence sources of disagreement is valuable; and that simply calculating a TDI or CP summary index may hide this detail. Therefore, if the TDI or CP is computed, we recommend that a Bland-Altman style plot of the between-device paired differences against the average is also constructed showing the raw mean bias and CAD, and we propose that this provides a sound way to assess agreement. In particular, any outliers or skewness in the data can be easily examined with respect to the CAD.

For both the limits of agreement and TDI methods it is important to remember that the calculated limits are only estimates (just as the CCC is a point estimate) and so uncertainty in the true values of these limits does exist [44]. Different samples from the overall population may produce different limits and a different TDI. In particular, when sample sizes are small the observed limits of agreement may be far away from the “true” limits of agreement due to finite sampling bias. This is why for statistical inference purposes, calculation of confidence bounds around the limits is often recommended or indeed calculation of separate prediction intervals [44, 45].

As a probability, the CP provides an intuitive measure of agreement that may be easily interpreted by users with almost any level of statistical sophistication. It also requires a clinically acceptable difference (CAD) to be pre-specified before it can be used, and so the resulting interpretation is directly related to the original measurement scale.

When applying the LoA, TDI, or CP methods, specification of the acceptable difference is required. It is important to note that this is a context-dependent decision that should be made by an expert that knows what it means for the devices to be practically equivalent. Whether differences between the devices tend to fall within the CAD or not depends on both the relative bias between them and their precisions. If the bias and imprecision is sufficiently small (as determined by the CAD) then for practical purposes the devices can be used interchangeably. This is an important decision, because an incorrectly specified CAD will lead to incorrect conclusions about the level of agreement.

Although the five agreement methods considered can be computed on the basis of similar linear modelling approaches, they deviate from one another according to: (i) which outcome is being measured (the differences or the raw observations), (ii) the main focus of the method (on comparison with the CAD or variance components), and (iii) how the variance components are used in the expressions of the indices. All methods may mask individual areas of disagreement in data and make implicit assumptions about aspects of the variability or modelled relationships. It is therefore important to look beyond the agreement indices themselves and examine the values and assumptions used to compute them. For example, it is easy to compute limits of agreement without adequately considering the variance components that were used to derive them or without considering the possibility of the devices having inherently different precisions. We recommend that researchers pre-specify the precise form of the statistical model they will use in a statistical analysis plan, since different models will lead to different agreement indices. For example we found that the results for some indices (e.g. CIA) changed depending on whether a device-activity interaction was assumed in the COPD example. The advantage of including this interaction is to be able to check if the agreement between the two devices varies from activity to activity or not. But this comes at the price of making the assumption of an extra additive term in the model that may or may not hold.

The need for confidence intervals alongside agreement limits is strongly indicated in the literature, and rightly so. However, we think it is equally – if not more – important to report the individual variance components (e.g. between-subject variance and within-subject variance) and bias estimates alongside agreement indices, because these will elucidate the source of disagreement. Additionally, it is important to be aware that disagreement between devices as observed in agreement indices may hide differences in the level of precisions and measurement error between the devices, and also reflect underlying mean biases that cannot be adequately modelled by absolute mean differences. This is why looking beyond the disagreement to the underlying causes is crucial in helping one to critically appraise agreement results.

All five methods rely on parametric assumptions. Non-parametric approaches to assessing agreement, such as the method by Perez-Jaume and Carrasco [30], are not often seen in the literature but should be considered; especially in cases where data is skewed or otherwise non-normal.

In our assessment of the agreement methods we implicitly assume that the sample size available is sufficient to achieve model convergence. In cases where the number of patients or repeated measurements is small, the methods may not perform well, and this is an avenue for future research.

The time at which measurements were taken was not considered to be clinically important in this study conditional on the other covariates, and so we did not adjust for time of measurement in the models. In other studies and settings however, time of measurement may be influential and will need to be accounted for in the models.

## Conclusions

Barnhart et al. (2016) provided pros and cons of several different agreement indices for both continuous and categorical data in a core lab setting and concluded that coverage probability is the preferred choice of assessing agreement in a core lab setting [11]. We agree that the coverage probability is an ideal choice to provide an easily interpretable summary index of agreement. However, we would not recommend providing just the coverage probability index, if there is any disagreement, because it may hide important nuances in the data, particularly relating to the source of the disagreement in applied clinical studies. We therefore recommend that researchers also construct a Bland-Altman plot (which also depicts the raw mean bias and clinically acceptable difference) to provide a helpful visual examination of the data [12] (see Fig. 3). The CCC should not be used as a sole agreement metric due to its potential to give biased results when the between-subject variability is high. Regardless of which agreement index one uses, we recommend summarizing the data graphically to provide preliminary insight into the agreement between the devices and to evaluate model assumptions.

## Supplementary information

**Additional file 1.**

**Additional file 2.**

## Data Availability

The dataset analysed in this study has already been made publically available by means of inclusion in a supplementary file in a previous publication [15]. There is no restriction for access, and it is available for download via: 10.1371/journal.pone.0168321.s003

## Abbreviations

ANOVA: Analysis Of Variance
BMI: Body Mass Index
CAD: Clinically acceptable difference
CCC: Concordance correlation coefficient
CI: Confidence interval
CIA: Coefficient of individual agreement
COPD: Chronic Obstructive Pulmonary Disease
CP: Coverage Probability
ICC: Intra-class correlation coefficient
IQR: Interquartile range
LoA: Limits of agreement
MSD: Mean squared deviation
PoA: Probability of agreement
Q-Q plot: Quantile-quantile plot
SD: Standard deviation
TDI: Total deviation index
