# Supplementary material for: Using multiple agreement methods for continuous repeated measures data: a tutorial for practitioners
Source: BMC Med Res Methodol. 2020 Jun 12;20:154. doi: 10.1186/s12874-020-01022-x (PMC7291585; doi:10.1186/s12874-020-01022-x)
Supplement: Supplementary file 1 — Additional file 1. [file 12874_2020_1022_MOESM1_ESM.pdf]

Supplementary Materials for "Using Multiple Agreement methods for Continuous Repeated Measures Data: A Tutorial for Practitioners"

Richard A. Parker, Charlie Scott, Vanda Inácio, and Nathaniel T. Stevens

In this supplementary file we provide further details about the Bland–Altman fixed effects approach and about the probability of agreement method mentioned in the main article, some exploratory plots of the COPD data, and diagnostic plots for the different linear mixed effect models fitted. The `R` code used to produce all results is also provided.

Limits of agreement for repeated measurements (Bland and Altman, 2007)

This approach is better explained by starting with the case where only one measurement is recorded per device on each subject. Let us denote the measurements recorded by each device on subject i by Y_{i1} and Y_{i2}, and let $D_i = Y_{i2} - Y_{i1}$ be the difference between (single measurements) of the two devices. Under the assumption that the differences are not related to the size of the measurements and that they are well approximated by a normal distribution, the 95% limits of agreement (LOA) are defined as

$$\mu_D \pm 1.96\sigma_D, \tag{1}$$

where $\mu_D = \mathbb{E}(D_i)$ and $\sigma_D^2 = \text{var}(D_i)$. The LOA can be estimated by replacing μ_D and σ_D^2 by their sample counterparts.

The limits of agreement approach was extended to the repeated measurements setup by Bland and Altman (2007). Due to the nature of our COPD example, we proceed with the assumption that the (unobserved) true value varies over the period of observation. The LOA are still defined as in (1), but with the exception that now the differences are defined in a slightly different way. Specifically, let D_{il} be the difference between the measurements of the two devices when subject i is performing activity l, i.e.,

$$D_{il} = Y_{i2l} - Y_{i1l}, \quad i = 1, \ldots, n, \quad l = 1, \ldots, m_i, \tag{2}$$

and now $\mu_D = \mathbb{E}(D_{il})$ and $\sigma_D^2 = \text{var}(D_{il})$. To estimate the mean of such differences, Bland and Altman (2007) suggested to use $\hat{\mu}_D = Y_{\cdot 2 \cdot} - Y_{\cdot 1 \cdot}$ averaging over indices i and l. The authors also propose to estimate the variance of the differences σ_D^2 via a one-way ANOVA model for D_{il}, namely

$$D_{il} = \mu_D + \tau_{Di} + \varepsilon_{Dil}, \tag{3}$$

where τ_{Di} represents the subject effect and ε_{Dil} is the independent random error within the subject for that pair of observations. It is worth mentioning that the model formulation in (3) can be easily adpated to deal with extra factors, such as the activity in our COPD example. Under this model, $\sigma_D^2 = \sigma_{D\tau}^2 + \sigma_{DW}^2$, where $\sigma_{D\tau}^2 = \text{var}(\tau_{Di})$ and $\sigma_{DW}^2 = \text{var}(\varepsilon_{Dil})$. Letting MSB_D and MSW_D denote the between-subject and within-subject mean squares from the ANOVA model in (3), the estimator for σ_D^2 is

$$\hat{\sigma}_D^2 = \frac{\text{MSB}_D - \text{MSW}_D}{\frac{(\sum_{i=1}^n m_i)^2 - \sum_{i=1}^n m_i^2}{(n-1)\sum_{i=1}^n m_i}} + \text{MSW}_D$$

where m_i denotes the number of observations on subject i ($i = 1, \ldots, n$). The first term on the right hand side is estimating $\sigma^2_{D\tau}$, while the second estimates σ^2_{DW}.

Probability of agreement

Stevens et al. (2017, 2018) developed the probability of agreement (PoA) method as an alternative to the limits of agreement approach, which has the advantage of taking into account two different types of bias and unequal precisions across devices. Proportional bias, where the magnitude of disagreement depends on the true value in each subject, is considered in addition to additive bias, and this information can be used to elucidate the different sources of disagreement if the devices disagree.

The PoA method simultaneously accounts for fixed bias, proportional bias, unequal precisions, and even non-constant measurement error (called heteroscedasticity) through an appropriately defined linear mixed structural model (see the model in Equation (4)). The probability of agreement itself is the probability that the difference between single measurements on the same subject by the two devices falls within a range that is deemed to be clinically acceptable, conditional on the true value of the outcome. Assuming that S represents the true value of the measurand, PoA is defined as

$$\theta(s) = \Pr(|Y_1 - Y_2| \leq \delta \mid S = s),$$

where Y_j represents a single reading by device j on a subject with true value s and $\pm\delta$ represents the interval within which differences in readings are considered practically negligible (i.e., the CAD).

This approach has the benefit of being intuitive and straightforward to interpret, regardless of one's level of statistical expertise; values close to 1 signify a high likelihood of differences between the devices being small enough to be clinically acceptable, while values close to 0 indicate that clinically acceptable differences are unlikely. Thus, large values of the PoA indicate agreement and small values indicate disagreement between devices. How large a PoA value needs to be to indicate sufficient agreement is user-dependent and, like the clinically acceptable difference, should be decided prior to executing the method comparison study. The PoA is similar in spirit to the coverage probability, but the PoA is conditional on the true value of the outcome and thus calculated and plotted across a range of plausible outcome values. In this way, the PoA clearly summarizes the dependence of agreement on the true outcome value. This is to be contrasted with the CP which provides a one-number summary which may or may not be applicable across the entire range of measurement.

Underlying the PoA methodology is the following linear mixed effect structural model

$$\begin{aligned} Y_{i1} &= S_i + \varepsilon_{i1}, \\ Y_{i2} &= \eta + \lambda S_i + \varepsilon_{i2}. \end{aligned} \tag{4}$$

Here $S_i \sim N(\mu, \sigma_s^2)$ is a random effect that describes the true value distribution and $\varepsilon_{ij} \sim N(0, \sigma_j^2)$ is a random effect that describes the measurement variation of device j. The fixed effects η and λ quantify the fixed and proportional bias, respectively. Note that it is implicitly assumed that the reference device is unbiased and so all inferences concerning bias are made from the second device relative to the reference. As in Stevens et al. (2017) the model in (4) allows for each device's measurement variation to be different, but it assumes that this variation is homoscedastic (constant across the distribution of true outcome values). However, Stevens et al. (2018) extend the PoA to the case in which one or both device's measurement variation is heteroscedastic.

Note that the probability of agreement methodology proposed in Stevens et al. (2017, 2018) assumed a balanced study, but by adapting the likelihood function and relevant `R` code we generalised the methodology here to account for the unbalanced case. Note however that model (4) and hence the PoA does not account for activity in the COPD example or interaction terms such as those included in model (2) in the main paper –

such random effects are subsumed by the error terms in (4). Although in principle the methodology could be adapted to include these random effects, as of yet it has not been and so this is a limitation of the methodology at present. As such, the probability of agreement has not been included in the main manuscript, but we still provide in the link below code that automates its computation, so that readers can play around with it.

`https://github.com/vandainacio/Comparison-of-Six-Agreement-Methods-for-Continuous-Repeated-Measures-Data`

References

Bland, J. M. and Altman, D. G. (2007). Agreement between methods of measurement with multiple observations per individual. *Journal of Biopharmaceutical Statistics*, 17(4):571–582.

Stevens, N. T., Steiner, S. H., and MacKay, R. J. (2017). Assessing agreement between two measurement systems: An alternative to the limits of agreement approach. *Statistical Methods in Medical Research*, 26(6):2487–2504.

Stevens, N. T., Steiner, S. H., and MacKay, R. J. (2018). Comparing heteroscedastic measurement systems with the probability of agreement. *Statistical Methods in Medical Research*, 27(11):3420–3435.

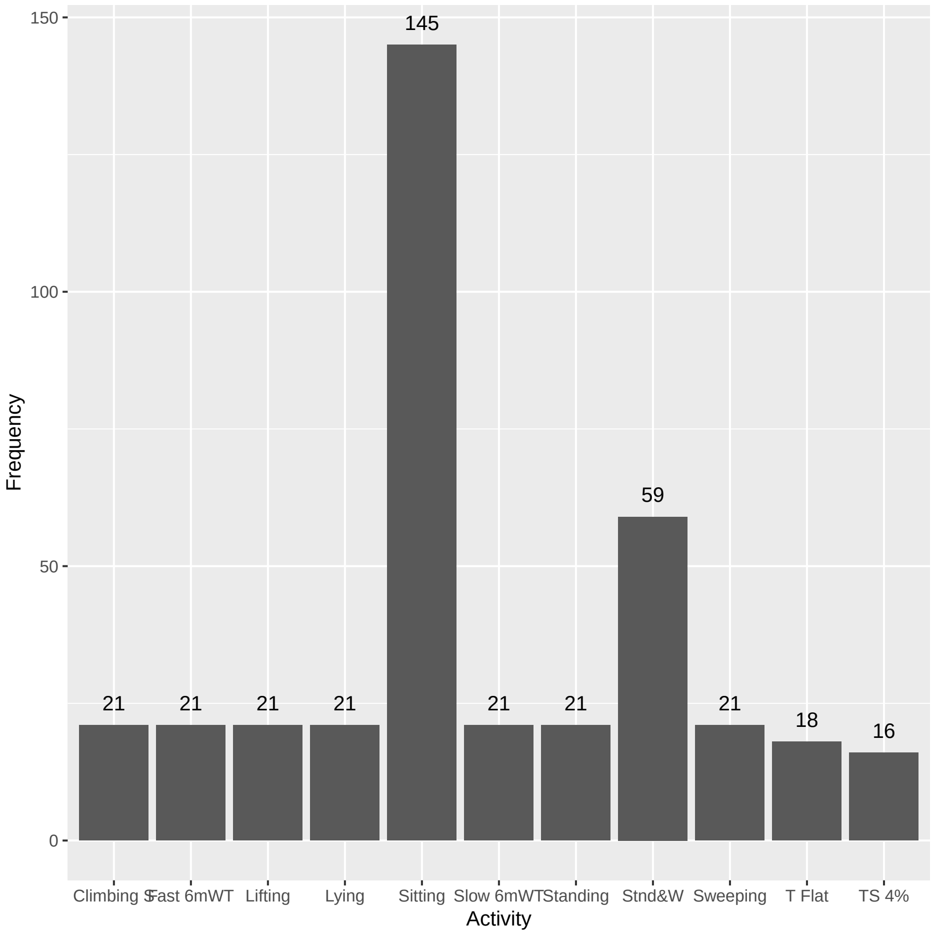

Figure 1: Barplot showing the frequency of each of the 11 activities over the 21 participants.

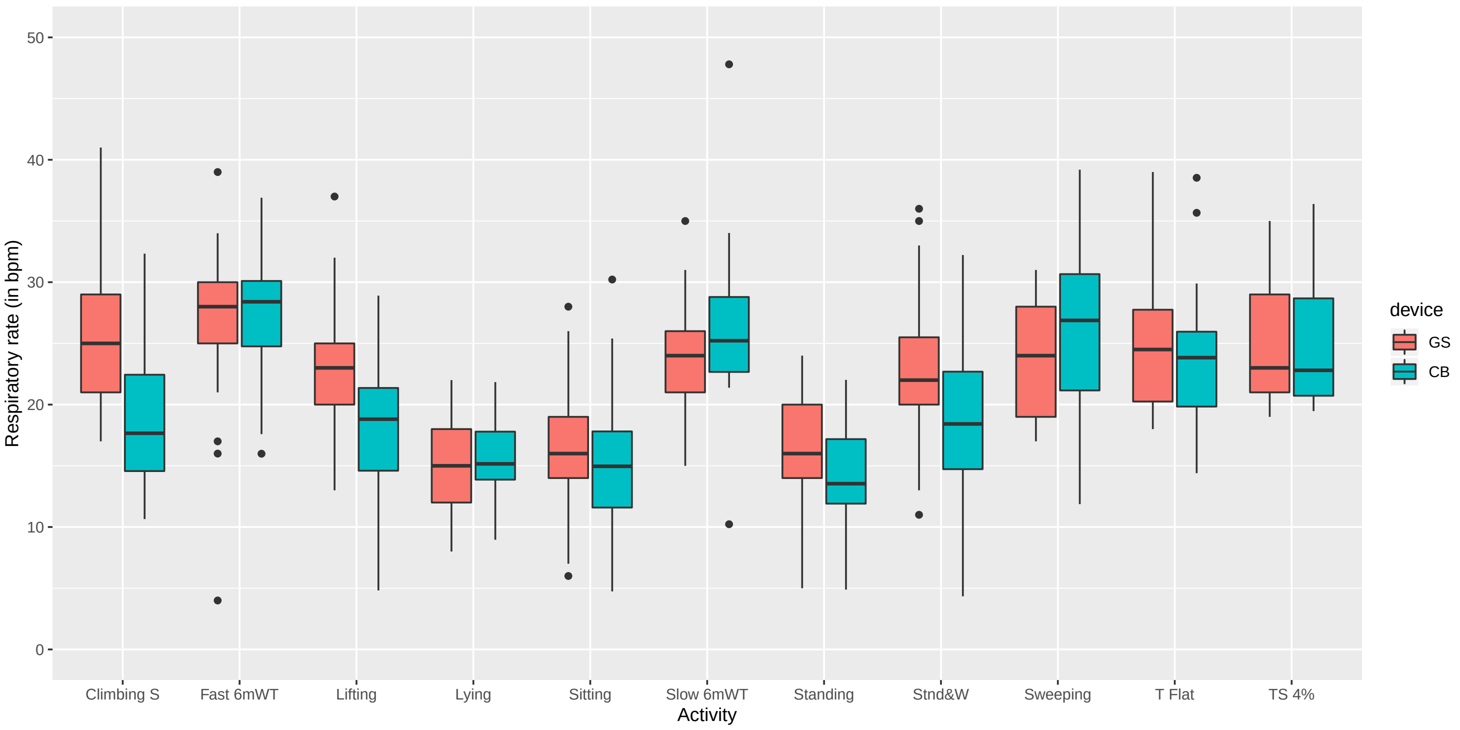

(a)

Figure 2: Boxplot of the respiratory rate measurements for each activity, when measured by the gold standard (GS) and by the chest-band device (CB).

Diagnostic plots for mixed models

In this section we show several plots that help (informally) checking the linear mixed model assumptions (listed in Box 1 of the main paper). All plots were produced with the aid of the `redres` package, which can be found at

`https://github.com/goodekat/redres`

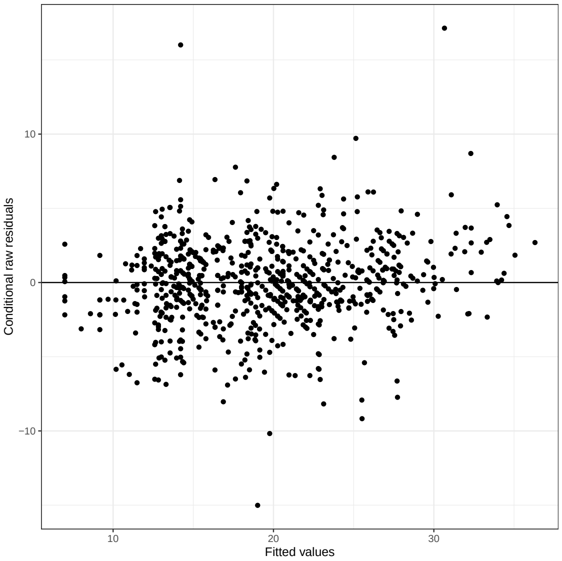

(a)

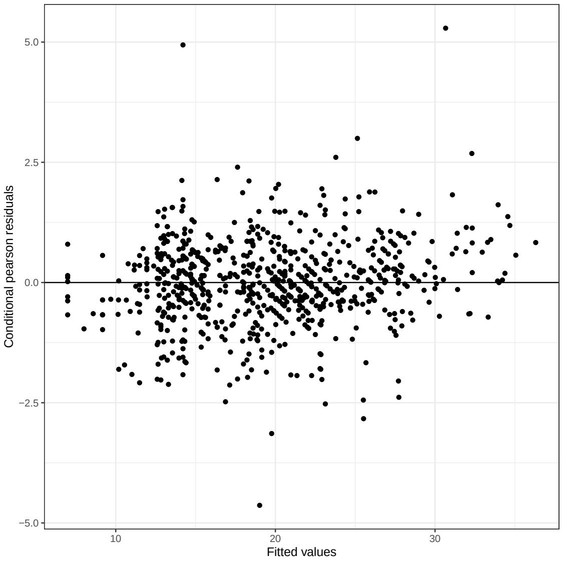

(b)

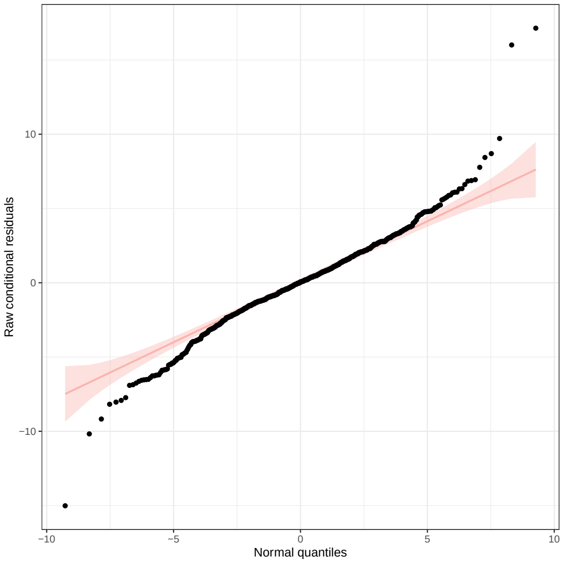

(c)

Figure 3: Diagnostics for model (2) in the main paper. (a) Conditional raw residuals versus the fitted values. (b) Conditional Pearson residuals versus the fitted values. (c) Normal Q-Q plot of the conditional raw residuals.

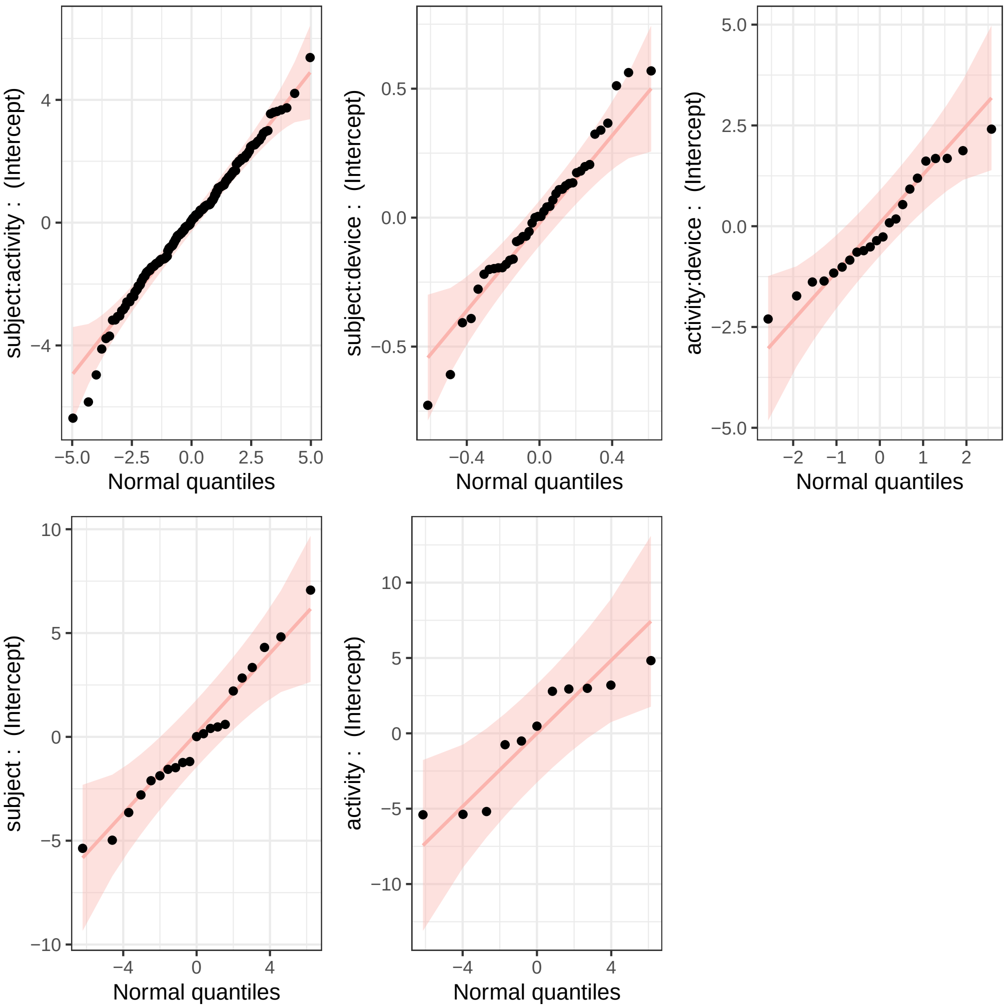

Figure 4: Diagnostics for model (2) in the main paper. Normal Q-Q plots for the random effects.

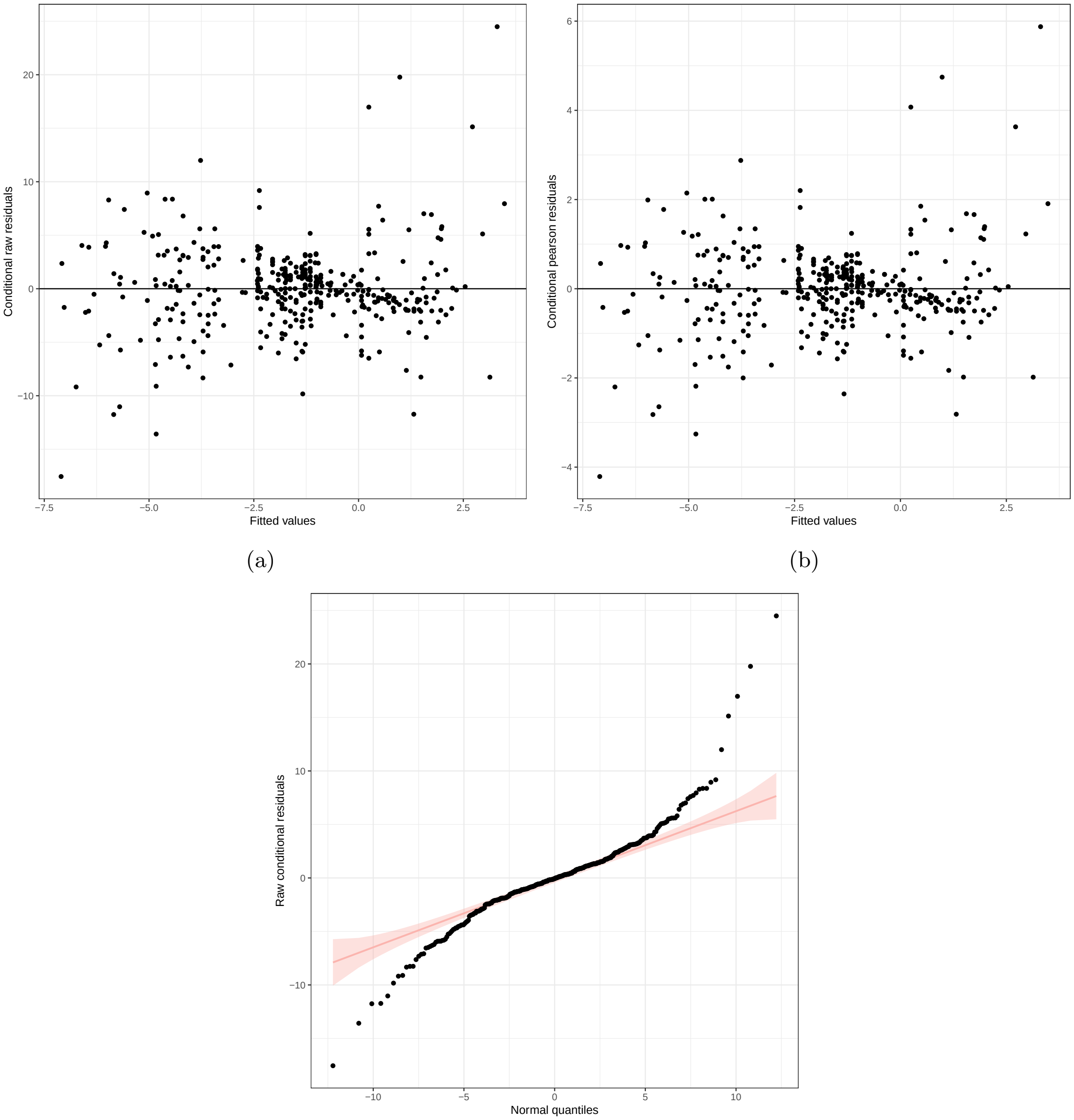

(a) (b)

(c)

Figure 5: Diagnostics for model (3) in the main paper. (a) Conditional raw residuals versus the fitted values. (b) Conditional Pearson residuals versus the fitted values. (c) Normal Q-Q plot of the conditional raw residuals.

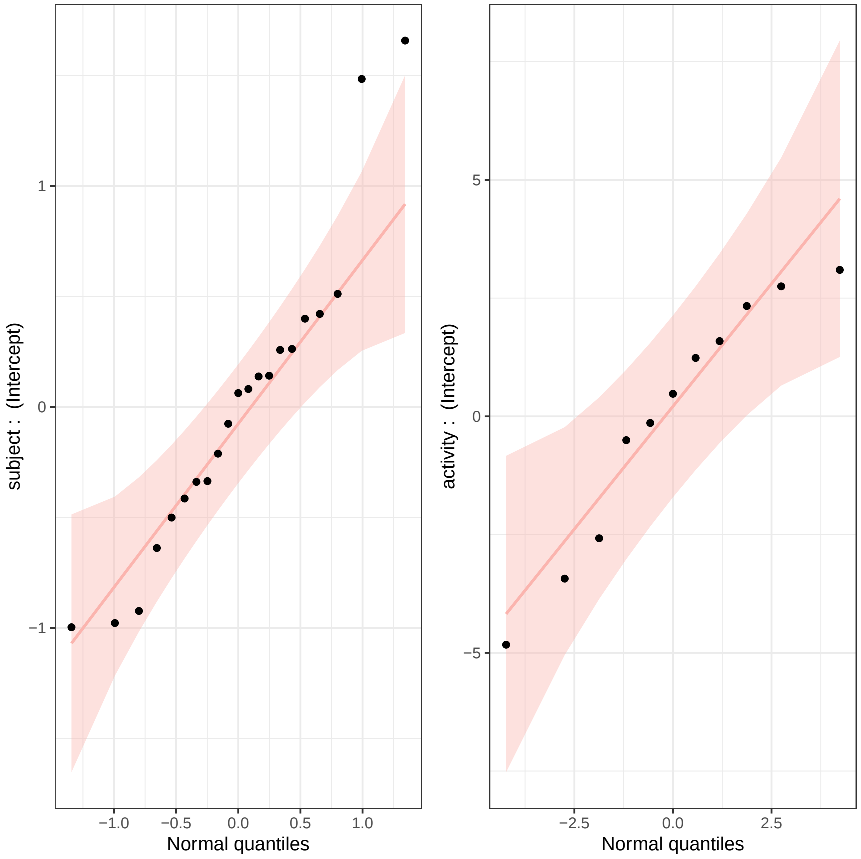

Figure 6: Diagnostics for model (3) in the main paper. Normal Q-Q plots for the random effects.

R code

```
require(lme4)

data <- read.table("copd.csv", sep = ",", fill = F, header = T)
y <- as.vector(c(data$RRox,data$RRcb))
subject <- as.vector(as.factor(c(data$PatientID,data$PatientID)))
device <- as.vector(as.factor(c(rep(1,385),rep(2,385))))
activity <- c(as.factor(data$Activity),as.factor(data$Activity))
copd <- data.frame(y, subject, device, activity)
copd$device <- as.factor(copd$device)
copd$activity <- as.factor(copd$activity)
copd$subject <- as.factor(copd$subject)

#Mixed effects model (1) in the main paper
res <- lmer(y ~ device + (1|subject) + (1|activity) +
              (1|subject:activity) + (1|subject:device) + (1|activity:device),
            data = copd,
            control = lmerControl(optimizer = "bobyqa")
            )
summary(res)

## Linear mixed model fit by REML ['lmerMod']
## Formula:
## y ~ device + (1 | subject) + (1 | activity) + (1 | subject:activity) +
##     (1 | subject:device) + (1 | activity:device)
##    Data: copd
## Control: lmerControl(optimizer = "bobyqa")
##
## REML criterion at convergence: 4330.4
##
## Scaled residuals:
##     Min      1Q  Median      3Q     Max
## -4.6334 -0.4680  0.0167  0.5096  5.2893
##
## Random effects:
##  Groups           Name        Variance Std.Dev.
##  subject:activity (Intercept)  6.0046  2.4504
##  subject:device   (Intercept)  0.3774  0.6144
##  activity:device  (Intercept)  3.6937  1.9219
##  subject          (Intercept) 11.3869  3.3745
##  activity         (Intercept) 16.5660  4.0701
##  Residual                     10.4983  3.2401
## Number of obs: 770, groups:
## subject:activity, 223; subject:device, 42; activity:device, 22; subject, 21; activity, 11
##
## Fixed effects:
##             Estimate Std. Error t value
## (Intercept)  22.1117     1.5718  14.068
## device2      -1.2826     0.8882  -1.444
##
```

```
## Correlation of Fixed Effects:
##         (Intr)
## device2 -0.283

beta2.est <- coef(summary(res))[2]
sigma2.alpha.est <- as.numeric(summary(res)$varcor[4])
sigma2.gamma.est <- as.numeric(summary(res)$varcor[5])
sigma2.alpha.gamma.est <- as.numeric(summary(res)$varcor[1])
sigma2.alpha.beta.est <- as.numeric(summary(res)$varcor[2])
sigma2.beta.gamma.est <- as.numeric(summary(res)$varcor[3])
sigma2.epsilon.est <- as.numeric(summary(res)$sigma)^2
phi2.beta.est <- beta2.est^2

#Concordance correlation coefficient
num_ccc <- sigma2.alpha.est + sigma2.gamma.est + sigma2.alpha.gamma.est
den_ccc <- sigma2.alpha.est + phi2.beta.est + sigma2.gamma.est +
           sigma2.alpha.gamma.est + sigma2.alpha.beta.est +
           sigma2.beta.gamma.est + sigma2.epsilon.est
CCC <- num_ccc/den_ccc
CCC

## [1] 0.676822

#Mean squared deviation
MSD <- (beta2.est^2) + 2*(sigma2.alpha.beta.est+sigma2.beta.gamma.est+sigma2.epsilon.est)
MSD

## [1] 30.78389

#Total deviation index
p <- 0.95
TDI <- qnorm((1+p)/2)*sqrt(MSD)
TDI

## [1] 10.87451

#Coverage probability
delta <- 5
CP <- 1-2*(1-pnorm(delta/sqrt(MSD)))
CP

## [1] 0.6325038

#Coefficient of individual agreement
CIA <- 2*sigma2.epsilon.est/MSD
CIA

## [1] 0.6820637

#Limits of agreement (mixed model approach--modelling the differences)
d <- copd$y[386:770]-copd$y[1:385]
subject <- as.factor(copd$subject[1:385])
```

```
activity <- as.factor(copd$activity[1:385])

res.diff <- lmer(d ~ (1|subject) + (1|activity),
                 control = lmerControl(optimizer = "bobyqa")
                 )

summary(res.diff)

## Linear mixed model fit by REML ['lmerMod']
## Formula: d ~ (1 | subject) + (1 | activity)
## Control: lmerControl(optimizer = "bobyqa")
##
## REML criterion at convergence: 2231.1
##
## Scaled residuals:
##     Min      1Q  Median      3Q     Max
## -4.2106 -0.4468 -0.0138  0.3886  5.8757
##
## Random effects:
##  Groups   Name        Variance Std.Dev.
##  subject  (Intercept)  0.9602  0.9799
##  activity (Intercept)  7.5652  2.7505
##  Residual             17.3753  4.1684
## Number of obs: 385, groups:  subject, 21; activity, 11
##
## Fixed effects:
##             Estimate Std. Error t value
## (Intercept)   -1.273      0.895  -1.422

totalsd <- sqrt(as.numeric(summary(res.diff)$varcor[1])+
                as.numeric(summary(res.diff)$varcor[2])+
                as.numeric(summary(res.diff)$sigma^2)
                )

res.diff.1 <- lmer(d ~ 1 + (1|subject),
                   control = lmerControl(optimizer = "bobyqa")
                  )

summary(res.diff.1)

## Linear mixed model fit by REML ['lmerMod']
## Formula: d ~ 1 + (1 | subject)
## Control: lmerControl(optimizer = "bobyqa")
##
## REML criterion at convergence: 2297.7
##
## Scaled residuals:
##     Min      1Q  Median      3Q     Max
## -4.7264 -0.4244  0.1472  0.3797  5.9935
##
## Random effects:
##  Groups   Name        Variance Std.Dev.
```

```
##  subject  (Intercept)  0.67    0.8186
##  Residual             22.36    4.7288
## Number of obs: 385, groups:  subject, 21
##
## Fixed effects:
##             Estimate Std. Error t value
## (Intercept)  -1.5960     0.3001  -5.317
```

```
meanb <- coef(summary(res.diff.1))[1]
meanb
```

```
## [1] -1.595991
```

```
alpha <- 0.05
z <- qnorm(1-alpha/2)

lcl <- meanb - z*totalsd
ucl <- meanb + z*totalsd
lcl; ucl
```

```
## [1] -11.57078
## [1] 8.378797
```

```
#limits of agreement (mixed model approach--raw data)
ll_raw <- beta2.est - z*sqrt(2*sigma2.alpha.beta.est + 2*sigma2.beta.gamma.est + 2*sigma2.epsilon.est)
ul_raw <- beta2.est + z*sqrt(2*sigma2.alpha.beta.est + 2*sigma2.beta.gamma.est + 2*sigma2.epsilon.est)
ll_raw; ul_raw
```

```
## [1] -11.86257
## [1] 9.297345
```

```
beta2.est
```

```
## [1] -1.282612
```

```
####bootstrap procedure
set.seed(123)
n <- 21
B <- 500
resb <- resdb <- resd1b <- list()

for(l in 1:B){

  ind <- sample(1:n, n, replace = TRUE)
  subject_boot <- list()

  for(j in 1:n){
    subject_boot[[j]] <- data[data$PatientID==ind[j],c(1,3,5,12)]
  }

  datab <- rbind(subject_boot[[1]], subject_boot[[2]], subject_boot[[3]], subject_boot[[4]],
                 subject_boot[[5]], subject_boot[[6]], subject_boot[[7]], subject_boot[[8]],
```

```r
                 subject_boot[[9]], subject_boot[[10]], subject_boot[[11]], subject_boot[[12]],
                 subject_boot[[13]], subject_boot[[14]], subject_boot[[15]], subject_boot[[16]],
                 subject_boot[[17]], subject_boot[[18]], subject_boot[[19]], subject_boot[[20]],
                 subject_boot[[21]]
                )

  yb <- as.vector(c(datab$RRox,datab$RRcb))

  aux <- c(rep(1,nrow(subject_boot[[1]])),rep(2,nrow(subject_boot[[2]])),
           rep(3,nrow(subject_boot[[3]])),rep(4,nrow(subject_boot[[4]])),
           rep(5,nrow(subject_boot[[5]])),rep(6,nrow(subject_boot[[6]])),
           rep(7,nrow(subject_boot[[7]])),rep(8,nrow(subject_boot[[8]])),
           rep(9,nrow(subject_boot[[9]])),rep(10,nrow(subject_boot[[10]])),
           rep(11,nrow(subject_boot[[11]])),rep(12,nrow(subject_boot[[12]])),
           rep(13,nrow(subject_boot[[13]])),rep(14,nrow(subject_boot[[14]])),
           rep(15,nrow(subject_boot[[15]])),rep(16,nrow(subject_boot[[16]])),
           rep(17,nrow(subject_boot[[17]])),rep(18,nrow(subject_boot[[18]])),
           rep(19,nrow(subject_boot[[19]])),rep(20,nrow(subject_boot[[20]])),
           rep(21,nrow(subject_boot[[21]]))
           )
  subjectb <- as.vector(as.factor(c(aux,aux)))
  deviceb <- as.vector(as.factor(c(rep(1,nrow(datab)),rep(2,nrow(datab)))))
  activityb <- c(as.factor(datab$Activity),as.factor(datab$Activity))
  db <- yb[(nrow(datab)+1):(2*nrow(datab))] - yb[1:nrow(datab)]
  copdb <- data.frame(yb, subjectb, deviceb, activityb, db)
  copdb$deviceb <- as.factor(copdb$deviceb)
  copdb$activityb <- as.factor(copdb$activityb)
  copdb$subjectb <- as.factor(copdb$subjectb)

  resb[[l]] <- lmer(yb ~ deviceb+(1|subjectb)+(1|activityb)+
                         (1|subjectb:activityb)+(1|subjectb:deviceb)+(1|activityb:deviceb),
                    data = copdb,
                    control = lmerControl(optimizer = "bobyqa")
                    )

  resdb[[l]] <- lmer(db ~ (1|subjectb) + (1|activityb),
                     data = copdb,
                     control = lmerControl(optimizer = "bobyqa")
                     )


  resd1b[[l]] <- lmer(db ~ 1 + (1|subjectb),
                      data = copdb,
                      control = lmerControl(optimizer = "bobyqa")
                      )
}

beta2.est.b <- numeric(B)
sigma2.alpha.est.b <- sigma2.gamma.est.b <- numeric(B)
sigma2.alpha.gamma.est.b <- sigma2.alpha.beta.est.b <- numeric(B)
sigma2.beta.gamma.est.b <- sigma2.epsilon.est.b <- numeric(B)
```

```
phi2.beta.est.b <- numeric(B)
CCCb <- TDIb <- CPb <- CIAb <- MSDb <- numeric(B)
meanbb <- totalsdb <- numeric(B)
lclb <- uclb <- ll_rawb <- ul_rawb <- numeric(B)

for(l in 1:B){
  beta2.est.b[l] <- coef(summary(resb[[l]]))[2]
  sigma2.alpha.est.b[l] <- as.numeric(summary(resb[[l]])$varcor[4])
  sigma2.gamma.est.b[l] <- as.numeric(summary(resb[[l]])$varcor[5])
  sigma2.alpha.gamma.est.b[l] <- as.numeric(summary(resb[[l]])$varcor[1])
  sigma2.alpha.beta.est.b[l] <- as.numeric(summary(resb[[l]])$varcor[2])
  sigma2.beta.gamma.est.b[l] <- as.numeric(summary(resb[[l]])$varcor[3])
  sigma2.epsilon.est.b[l] <- as.numeric(summary(resb[[l]])$sigma)^2
  phi2.beta.est.b[l] <-  beta2.est.b[l]^2
  num_ccc.b <- sigma2.alpha.est.b[l] + sigma2.gamma.est.b[l] + sigma2.alpha.gamma.est.b[l]
  den_ccc.b <- sigma2.alpha.est.b[l] + phi2.beta.est.b[l] +
               sigma2.gamma.est.b[l] + sigma2.alpha.gamma.est.b[l] +
               sigma2.alpha.beta.est.b[l] + sigma2.beta.gamma.est.b[l] +
               sigma2.epsilon.est.b[l]
  CCCb[l] <- num_ccc.b/den_ccc.b
  MSDb[l] <- (beta2.est.b[l]^2) +
    2*(sigma2.alpha.beta.est.b[l]+sigma2.beta.gamma.est.b[l]+sigma2.epsilon.est.b[l])
  TDIb[l] <- qnorm((1+p)/2)*sqrt(MSDb[l])
  CPb[l] <- 1-2*(1-pnorm(delta/sqrt(MSDb[l])))
  CIAb[l] <- 2*sigma2.epsilon.est.b[l]/MSDb[l]
  totalsdb[l] <- sqrt(as.numeric(summary(resdb[[l]])$varcor[1])+
                      as.numeric(summary(resdb[[l]])$varcor[2])+
                      as.numeric(summary(resdb[[l]])$sigma^2)
                      )
  meanbb[l] <- coef(summary(resd1b[[l]]))[1]
  lclb[l] <- meanbb[l] - z*totalsdb[l]
  uclb[l] <- meanbb[l] + z*totalsdb[l]
  ll_rawb[l] <- beta2.est.b[l] - z*sqrt(2*sigma2.alpha.beta.est.b[l] +
                                     2*sigma2.beta.gamma.est.b[l] + 2*sigma2.epsilon.est.b[l])
  ul_rawb[l] <- beta2.est.b[l] + z*sqrt(2*sigma2.alpha.beta.est.b[l] +
                                        2*sigma2.beta.gamma.est.b[l] + 2*sigma2.epsilon.est.b[l])

}

MSD; quantile(MSDb, c(0.025,0.975))

## [1] 30.78389
##     2.5%    97.5%
## 22.98767 41.65718

CCC; quantile(CCCb, c(0.025,0.975))

## [1] 0.676822
##      2.5%     97.5%
## 0.5960483 0.7208343

TDI; quantile(TDIb, c(0.025,0.975))
```

```
## [1] 10.87451
##      2.5%     97.5%
##  9.397085 12.650072
```

```
CP; quantile(CPb, c(0.025,0.975))
```

```
## [1] 0.6325038
##      2.5%     97.5%
## 0.5614741 0.7029882
```

```
CIA; quantile(CIAb, c(0.025,0.975))
```

```
## [1] 0.6820637
##      2.5%     97.5%
## 0.5653724 0.7526850
```

```
lb <- quantile(lclb, c(0.025, 0.975))
lcl; lb
```

```
## [1] -11.57078
##       2.5%      97.5%
## -13.515309  -9.937825
```

```
ub <- quantile(uclb, c(0.025, 0.975))
ucl; ub
```

```
## [1] 8.378797
##      2.5%     97.5%
##  6.372063 10.690036
```

```
meanbbq <- quantile(meanbb, c(0.025, 0.975))
meanb; meanbbq
```

```
## [1] -1.595991
##       2.5%      97.5%
## -2.1307454 -0.9747718
```

```
lrawb <- quantile(ll_rawb, c(0.025, 0.975))
ll_raw; lrawb
```

```
## [1] -11.86257
##      2.5%     97.5%
## -13.73020 -10.33243
```

```
urawb <- quantile(ul_rawb, c(0.025, 0.975))
ul_raw; urawb
```

```
## [1] 9.297345
##      2.5%     97.5%
##  7.372611 11.421008
```

```
b2b <- quantile(beta2.est.b, c(0.025, 0.975))
beta2.est; b2b
```

```
## [1] -1.282612
##       2.5%      97.5%
## -1.8945034 -0.4927148

m <- (copd$y[1:385] + copd$y[386:770])/2

plot(m, d, xlab = "Average", ylab = "Difference", ylim = c(-30,30))
abline(h=ucl, lwd = 2, lty = 2)
abline(h=ub[1], lwd = 3, lty = 3)
abline(h=ub[2], lwd = 3, lty = 3)
abline(h=lcl, lwd = 2, lty = 2)
abline(h=lb[1], lwd = 3, lty = 3)
abline(h=lb[2], lwd = 3, lty = 3)
abline(h=0, lwd = 2)
abline(h=meanb, lwd = 2, lty = 2)
abline(h=meanbbq[1], lwd = 3, lty = 3)
abline(h=meanbbq[2], lwd = 3, lty = 3)
```

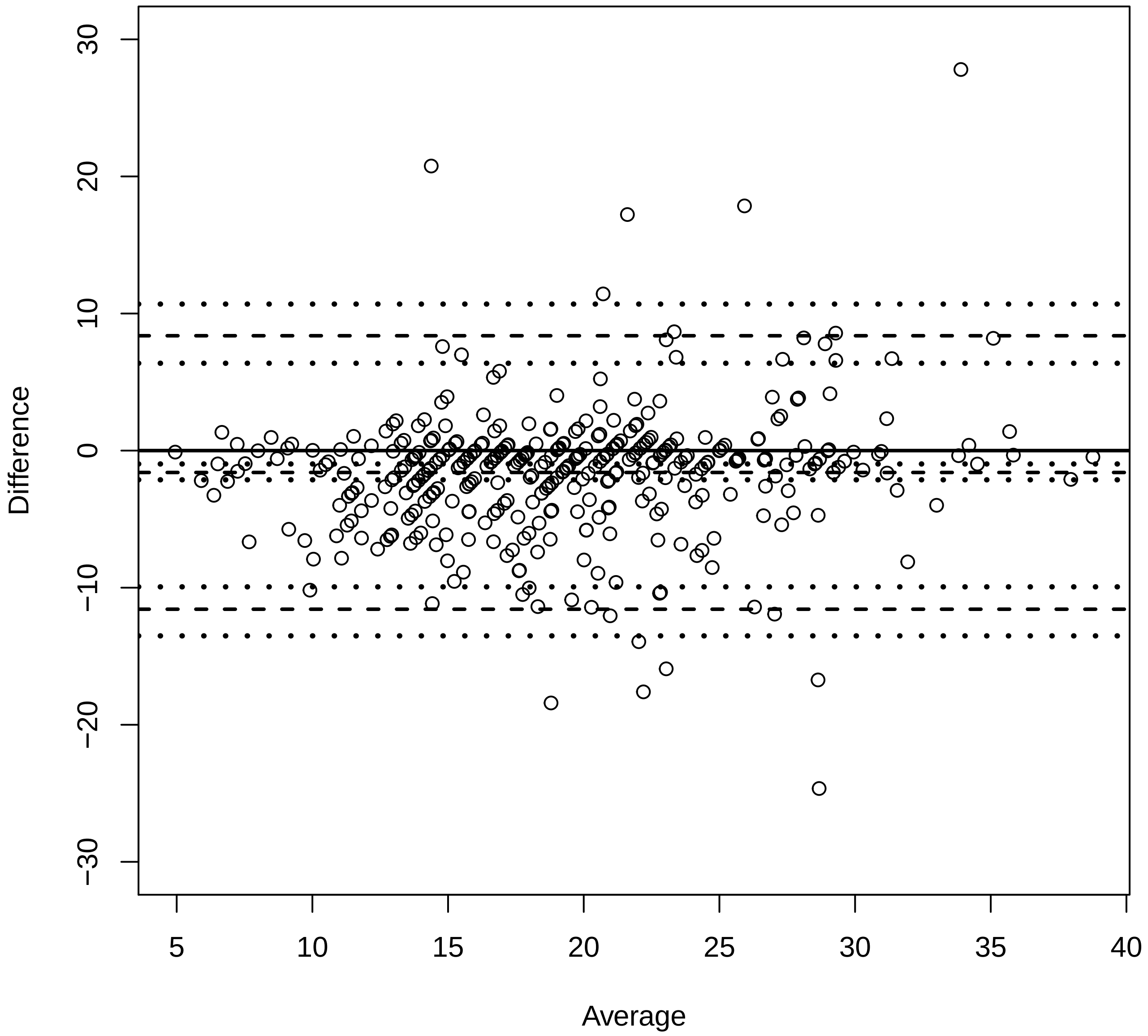
Difference
Average
30
20
10
0
−10
−20
−30
5
10
15
20
25
30
35
40
